# Temperature-Dependent Selection of Reaction Pathways, Reactive Species, and Products during Postsynthetic Selenization of Copper Sulfide Nanoparticles

**DOI:** 10.1021/acs.chemmater.3c01772

**Published:** 2023-10-20

**Authors:** Brandon Hole, Qi Luo, Ronald Garcia, Wanrui Xie, Eli Rudman, Chi Loi Thanh Nguyen, Diya Dhakal, Haley L. Young, Katherine L. Thompson, Auston G. Butterfield, Raymond E. Schaak, Katherine E. Plass

**Affiliations:** † Department of Chemistry, Franklin & Marshall College, Lancaster, Pennsylvania 17604, United States; ‡ Department of Chemistry, The Pennsylvania State University, University Park, Pennsylvania 16802, United States; § Department of Chemistry, Department of Chemical Engineering, Materials Research Institute, The Pennsylvania State University, University Park, Pennsylvania 16802, United States

**Keywords:** post-synthetic transformation, copper chalcogenide, nanoheterostructure, anion exchange, seeded growth, nanoparticle dissolution, nanoparticle growth

## Abstract

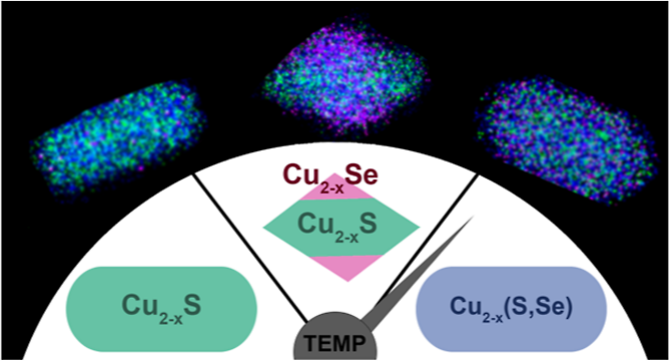

Rational design of elaborate, multicomponent nanomaterials is important for the development of many technologies such as optoelectronic devices, photocatalysts, and ion batteries. Combination of metal chalcogenides with different anions, such as in CdS/CdSe structures, is particularly effective for creating heterojunctions with valence band offsets. Seeded growth, often coupled with cation exchange, is commonly used to create various core/shell, dot-in-rod, or multipod geometries. To augment this library of multichalcogenide structures with new geometries, we have developed a method for postsynthetic transformation of copper sulfide nanorods into several different classes of nanoheterostructures containing both copper sulfide and copper selenide. Two distinct temperature-dependent pathways allow us to select from several outcomes—rectangular, faceted Cu_2–*x*_S/Cu_2–*x*_Se core/shell structures, nanorhombuses with a Cu_2–*x*_S core, and triangular deposits of Cu_2–*x*_Se or Cu_2–*x*_(S,Se) solid solutions. These different outcomes arise due to the evolution of the molecular components in solution. At lower temperatures, slow Cu_2–*x*_S dissolution leads to concerted morphology change and Cu_2–*x*_Se deposition, while Se-anion exchange dominates at higher temperatures. We present detailed characterization of these Cu_2–*x*_S–Cu_2–*x*_Se nanoheterostructures by transmission electron microscopy (TEM), powder X-ray diffraction, energy-dispersive X-ray spectroscopy, and scanning TEM–energy-dispersive spectroscopy. Furthermore, we correlate the selenium species present in solution with the roles they play in the temperature dependence of nanoheterostructure formation by comparing the outcomes of the established reaction conditions to use of didecyl diselenide as a transformation precursor.

## Introduction

Postsynthetic transformations have been used to create numerous multicomponent metal chalcogenide nanoparticles through processes such as cation and anion exchange, seeded growth, etching, shape changes, and oxidation.^1−6^ This control over process, composition, and geometry has enabled optimization of nanoheterostructures for various applications. Examples include photocatalytic hydrogen production based on the geometry of CdSe–CdS–Pt heterostructures,^7^ optimization of quantum dot inks for photovoltaics,^8^ and maximization of near-infrared emission.^6^ The ability to select among these different postsynthetic transformation pathways is, however, a crucial aspect of rational design that is still in early development. We envision being able to craft a postsynthetic transformation pathway where one nanoparticle synthon is transformed to another to achieve a desired multicomponent particle with tunability and location-specific placement (regioselectivity). To do this, we need clear delineation of the conditions under which similar transformation steps operate, as well as an understanding of how they can be selectively accessed. Ion exchange and directed growth processes, which are two of the most common methods for making heterostructured nanoparticles, often employ similar reagents and reaction conditions and thus can be in direct competition with one another. For example, directed growth of CdS arms on a range of seeds occurs simultaneously with cation exchange of the core.^6,9^ Selection between cation exchange and metal deposition on Cu_2–*x*_Se follows design rules based on the compatibility of the lattice structures.^10,11^ A particularly complex case reviewed by Kolny-Olesiak^12^ details how copper sulfide precursors result in numerous hybrid nanostructures by acting as catalysts, seeds, or through cation exchange.

Here, we present the synthesis of three different Cu_2–*x*_S–Cu_2–*x*_Se nanoheterostructures that expands the existing library of Cu_2–*x*_S–Cu_2–*x*_Se structures and provides new synthetic tools for creation of nanoheterostructures with different anionic components. Multiple-anion nanoheterostructures are a powerful platform for tuning optoelectronic properties, as recently demonstrated by CdSe/CdS/CdTe core/barrier/crown structures.^13^ In this structure, the much lower valence band on CdS encourages charge separation between the CdSe core and CdTe crown and enables photon up-conversion. Typically, such multiple-anion structures involve the growth of a uniform shell or facet-directed growth based on the crystal structure of the initial seed. An array of Cu_2–*x*_S–Cu_2–*x*_Se nanoheterostructures have been created through cation exchange of CdS–CdSe structures obtained by such seeded growth including platelets,^14^ dot-in-rod structures where rods grow from wurtzite seed faces,^15^ and branched structures where arms grow from zinc blende seeds.^16,17^ The system here provides new geometries that do not require growth of the overall particle size, including a nanorhombus structure with largely exposed heterojunctions. The Cu_2–*x*_S–Cu_2–*x*_Se nanoparticle system has received considerable attention for their promising properties and as useful starting materials for cation exchange.^15,18,19^ These copper chalcogenides have been studied as solid electrolytes for Li^+^ batteries,^20,21^ thermoelectrics,^22,23^ photothermal agents,^24^ NIR plasmonics,^25^ and for pollutant reduction.^26,27^ The phase-selective synthesis of Cu_2–*x*_S^28,29^ and Cu_2–*x*_Se^30−32^ nanoparticles has been studied in detail. Cu_2–*x*_(S,Se) particles, which have mixed sulfur and selenium as a solid solution rather than as a phase-segregated heterostructure, have been made through several routes, including direct synthesis of hexagonal and cubic alloys,^20,33^ cation exchange of Cd(S,Se),^34^ and oxidation of core/shell Cu_2–*x*_Se/Cu_2–*x*_S particles.^35^ Other approaches to consider for making Cu_2–*x*_S–Cu_2–*x*_Se heterostructures involve direct seeded growth and anion exchange. Cu_2–*x*_S with various phases and shapes has been used to seed growth of a wide array of additional metal sulfides.^36^ Anion exchange with sulfide or selenide starting materials has rarely been demonstrated due to the low diffusion rates for large anions. Anion exchange in general is usually accompanied by significant shape changes and the introduction of Kirkendall voids.^37−40^ A Te^2–^ exchange process transforming Cu_2–*x*_S nanorods to Cu_2–*x*_Te nanorods without formation of Kirkendall voids was recently discovered.^41^ Here, partial exchange resulted in various regioselectivities, including a single core–shell and a double core/shell structure. This method was extended to create Cu_2–*x*_(Se,Te) from Cu_2–*x*_Se.^42^ This current work adds a new example of anion exchange on a sulfide to incorporate selenide and offers a pathway to composition-morphology control that is inaccessible via direct or seeded growth.

We address the deficiencies in rational design of postsynthetic transformations by revealing the molecular basis of selection between two competing postsynthetic pathways—directed growth and anion exchange. This provides insights into the complex solution chemistry that affects reaction pathways as well as a new tool for introducing a second anion into an existing particle. When Cu_2–*x*_S nanorods in oleylamine are injected into a mixture of dodecanethiol, Se, and octadecene, we find that each component has complicated behaviors as well as interactivity with the other components. Dodecanethiol is known to play various roles in the synthesis, surface chemistry, and transformations of copper sulfide.^43−45^ Thiols can alter the shape and phase of Cu_1.8_S nanorods.^45^ Thiols can reduce Se^46^ and SeO_2_,^47^ which form alkylammonium selenides with oleylamine. Octadecene can polymerize during synthesis^48^ or reduce chalcogens.^49^ Se-octadecene is a highly reactive metal selenide precursor,^50^ as are various Se-alkyl species that may form in solution.^31,32,51,52^ In addition to this complex solution chemistry, Cu_2–*x*_S_*y*_Se_1–*y*_ nanoparticles (instead of Cu_2–*x*_S–Cu_2–*x*_Se heterostructures) can be synthesized from combinations of these same reagents.^33^ All of these possible reaction pathways and outcomes compete, leading to the potential for complex behavior and also underscoring the importance of identifying and understanding the chemistry that is in play during the reactions.

Here, we describe a postsynthetic transformation system in which deposition coupled with morphology change is in close competition with anion exchange and the route taken can be selectively targeted via tuning reaction temperature. Injection of Cu_2–*x*_S nanorods in oleylamine into a solution of Se, dodecanethiol (ddt), and octadecene at different temperatures yields three new, different Cu_2–*x*_S–Cu_2–*x*_Se nanoheterostructures with distinctive shapes and regioselectivities of Cu_2–*x*_S and Cu_2–*x*_Se. This adds to the arsenal of transformation techniques that can be employed for rational design of elaborate multicomponent nanoparticles and provides insights into the mechanism of deposition versus ion exchange.

## Experimental Section

### Chemicals

The reagents used for the synthesis of roxbyite nanoparticles and subsequent Se-transformation include copper nitrate trihydrate [Cu(NO_3_)_2_·3H_2_O, 99.95%], sulfur powder (99.98%), selenium powder, trioctylphosphine oxide (≥90%), oleylamine (technical grade, 70%), 1-octadecene (technical grade, 90%), *tert*-dodecyl mercaptan (*t*-ddt) (mixture of isomers, 98.5%), and 1-dodecanethiol (ddt) (≥98%). All solvents used for precipitation and washing of the nanoparticles, including ethanol, isopropyl alcohol, toluene, hexane, and acetone, were of analytical grade. Unless specified, all reagents were purchased from Sigma-Aldrich and used as received.

### General Safety Concerns

The synthetic methods are performed under air-free conditions at elevated temperatures using high-boiling-point solvents. As such, care should be taken to ensure the proper monitoring and handling. For example, burns have been reported from exposure to high-temperature oleylamine.^53^ The synthesis of didecyl diselenide resulted in red selenium buildup in the bubbler, suggesting the potential for release of volatile, toxic selenium compounds. Proper containment and venting were ensured. This synthesis also requires sodium borohydride, which reacts vigorously with ethanol and water to release flammable H_2_ gas. Care must be taken to avoid overpressurization and to avoid fire. The safety data sheets for all chemicals used in the reactions should be reviewed, and proper personal protective equipment should be used. These reactions have the potential to evolve toxic gases and, as such, should be handled in a properly functioning fume hood.

### Standard Reaction Vessel Setup

Each of the following procedures utilizes either a standard Schlenk line setup or an Ar gas manifold. Each dried, three-necked, round-bottom flask was equipped with a magnetic stir bar, a reflux condenser with a glass adaptor connected either to the Schlenk line or to a bubbler, a thermocouple inserted through a silicone septum, and a second septum with a needle connected to the Ar gas. The temperature was controlled by heating mantles on magnetic stir plates.

### Synthesis of Copper Sulfide Nanorods

Cu_2–*x*_S nanorods were synthesized in 40% yield (based on a calculation using Cu(NO_3_)_2_·3H_2_O as the limiting reagent, assuming formation of Cu_1.8_S nanorods without including the mass of the ligands) using an adaptation^41^ of previously published procedures.^54,55^

### Selenium-Transformation Procedure

The Se-transformation procedure was modeled on a Te-exchange procedure initially published by Saruyama et al.^39^ and adapted in Garcia-Herrera et al’s^41^ study. A ddt-Se solution was prepared by adding Se powder (0.3 mmol, 0.02370 g) in ddt (2 mL) and octadecene (10 mL) to a 25 mL three-neck flask. This solution was held at varying temperatures (185, 200, or 260 °C) for 15–20 min, resulting in dissolution of the Se metal. A suspension of Cu_2–*x*_S nanorods in hexane (4 mL of ∼5 mg/mL to give ∼20 mg) was air-dried in a septum-capped vial, and then oleylamine (4 mL) was added. The vial was then placed under Ar blanket by purging for 5 min. The vial was parafilmed and sonicated for ∼10 min to disperse particles. The nanorod/oleylamine suspension was then swiftly injected into the ddt-Se solution at the desired temperature. The solution was left to exchange for the desired temperature and time (10 min −2 h). After the reaction, the reaction flask was cooled to room temperature and ethanol was added (20 mL) and centrifuged (6000 rpm for 5 min in 50 mL plastic centrifuge tubes) to isolate the nanorods as black precipitates. A second and third wash was carried out with a 4:1 ratio of ethanol/hexane. The precipitates were readily suspended in nonpolar solvents such as hexane and toluene. This procedure was reproduced reliably by several students at both Franklin & Marshall College and the Pennsylvania State University. If the temperature is not carefully controlled, the particles can dissolve at temperatures slightly above 260 °C and reprecipitate as cubic Cu_2–*x*_Se.

### Selenium-Transformation Procedure with Didecyl Diselenide

Didecyl diselenide was synthesized using published procedures with slight modifications.^31,56^ A 25 mL three-neck flask equipped with a reflux condenser, bubbler, stir bar, silicon septa, and a thermocouple was flushed with argon. Selenium powder (0.465 g, 5.89 mmol) was added, followed by sodium borohydride (0.490 g, 12.8 mmol). Anhydrous ethanol was added (3.75 mL) slowly to keep the temperature constant. The reaction mixture was stirred for 20 min. Afterward, additional selenium (0.465 g, 5.89 mmol) was added. The reaction was cooled to room temperature, followed by 20 min stirring. The flask was then heated to 70 °C and allowed to stir for 20 min, resulting in a dark red solution. The reaction mixture was again cooled to room temperature, and 1-bromodecane (3.25 mL, 13.5 mmol) and tetrahydrofuran (14 mL) were added dropwise over a few minutes. Reaction mixture was then allowed to stir for 48 h at room temperature. Phases were separated by using diethyl ether. The organic layer was washed with DI water. The combined organic layers were dried in MgSO_4_. The product was recrystallized using heptane and isopropyl alcohol, and its identity was verified using ^1^H and ^77^Se NMR. ^1^H NMR (400 MHz, CDCl_3_): δ 2.92 (t, 4H), 1.73 (q, 4H), 1.27 (m, 36H), 0.88 (t, 3H) ppm. ^77^Se (76 MHz, CDCl_3_): δ 307.6 ppm.

Selenium transformation was carried out using didecyl diselenide by adapting the standard procedure as follows. Didecyl diselenide (0.0745 g, 0.15 mmol) was dissolved in 10 mL of octadecene and added to the standard reaction setup and flushed with Ar. The reaction mixture was heated to either 185 or 260 °C, followed by injection of suspended nanorods (20 mg) in 4 mL of oleylamine. Reaction mixture was stirred for 90 min at constant temperature and then cooled to room temperature. Ethanol (20 mL) was added, and the solution was centrifuged (5 min at 6000 rpm). Washing was repeated using a 4:1 ethanol/hexane ratio of ethanol to hexane.

### Evaluation of Solution Species

A series of reaction mixtures were developed to identify potential reactive species formed in solution. The temperatures at which the solutions were tested are in accordance with selenium-exchange protocols (185, 200, and 260 °C). At each temperature, two groups of reagents were integrated. In the first group, selenium (0.0237 g, 0.300 mmol), 1-dodecanethiol (2 mL), and octadecene (10 mL) were incorporated. In the second group, only 1-dodecanethiol and octadecene were incorporated in the same proportions. Both groups were heated at a respective temperature for 120 min.

### Characterization

#### Powder X-ray Diffraction

After nanoparticles were cleaned and resuspended in hexane, they were cast onto glass slides and allowed to dry. The powder X-ray diffraction (PXRD) data were collected using a PANalytical X’Pert Pro X-ray diffractometer with Cu Kα radiation. The samples were scanned with 10 repetitions at a current of 40 mA and a voltage of 45 kV. Using PANalytical HighScore Plus software, the 10 scans were summed and patterns were compared from the ICDD database to determine the structure of the nanoparticles. Crystal structure and powder diffraction simulations were performed using CrystalMaker and CrystalDiffract from CrystalMaker Software Ltd., Oxford, England.

#### Transmission Electron Microscopy

Samples were prepared by placing a drop of nanoparticles suspended in hexane or toluene on a Au- or Ni-supported ultrathin carbon-coated transmission electron microscopy (TEM) grid (Electron Microscopy Sciences). TEM images of the particles and their average sizes were obtained using one of the two microscopes, a Delong Instruments LVEM25 Low-Voltage TEM at Franklin & Marshall College or the Talos TEM at the Materials Characterization Laboratory at the Pennsylvania State University. LVEM25 was operated under 25 kV with the Zyla 5.5 Scientific CMOS camera with appropriate alignments and enhancements. ImageJ software was used to analyze the TEM images.

#### Scanning Electron Microscopy/Energy-Dispersive X-ray Spectroscopy

Nanoparticles previously cast onto the PXRD slides were immobilized on a small piece of conductive carbon tape and affixed to a metal stub. Scanning electron microscopy (SEM) and energy-dispersive spectroscopy (EDS) of the sample were then carried out at 20 kV with an Evex Mini-SEM.

#### HAADF STEM/EDS Mapping

Samples were prepared by placing a drop of nanoparticles suspended in hexane or toluene on a Ni- or Au-supported ultrathin carbon-coated TEM grid (Electron Microscopy Sciences). The microscope employed was an FEI Talos F200X with a SuperX EDS at 200 kV in the Materials Characterization Laboratory at Pennsylvania State University accessed remotely. ImageJ software was used to analyze the high-resolution (HR)-TEM images. Bruker ESPRIT 2 software was used to interpret the scanning TEM (STEM)-EDS elemental map data.

#### X-ray Photoelectron Spectroscopy

X-ray photoelectron spectroscopy (XPS) experiments were performed using a Physical Electronics VersaProbe III instrument equipped with a monochromatic Al Kα X-ray source (*h*ν = 1486.6 eV) and a concentric hemispherical analyzer. Charge neutralization was performed using both low-energy electrons (<5 eV) and argon ions. The binding energy axis was calibrated using sputter-cleaned Cu (Cu 2p_3/2_ = 932.62 eV and Cu 3p_3/2_ = 75.1 eV) and Au foils (Au 4f_7/2_ = 83.96 eV). Peaks were charge referenced to the CH_*x*_ band in the carbon 1s spectra at 284.8 eV. Measurements were made at a takeoff angle of 45° with respect to the sample surface plane. This resulted in a typical sampling depth of 3–6 nm (95% of the signal originated from this depth or shallower). Quantification was done using instrumental relative sensitivity factors that account for the X-ray cross section and inelastic mean free path of the electrons. On homogeneous samples, major elements (>5 atom %) tend to have standard deviations of <3%, while minor elements can be significantly higher. The analysis size was ∼200 μm in diameter.

#### NMR Characterization of Didecyl Diselenide and Reaction Mixtures

NMR spectra were obtained with a Varian INOVA 500 multinuclear Fourier transform NMR spectrometer with frequencies of 499.7 MHz for ^1^H and 76 MHz for ^77^Se. Spectra were processed by using MestReNova. Spectra taken in CDCl_3_ were referenced to the solvent (CDCl_3_ = 7.26 ppm) as an internal standard.

## Results and Discussion

### Overview

**Figure 1:**
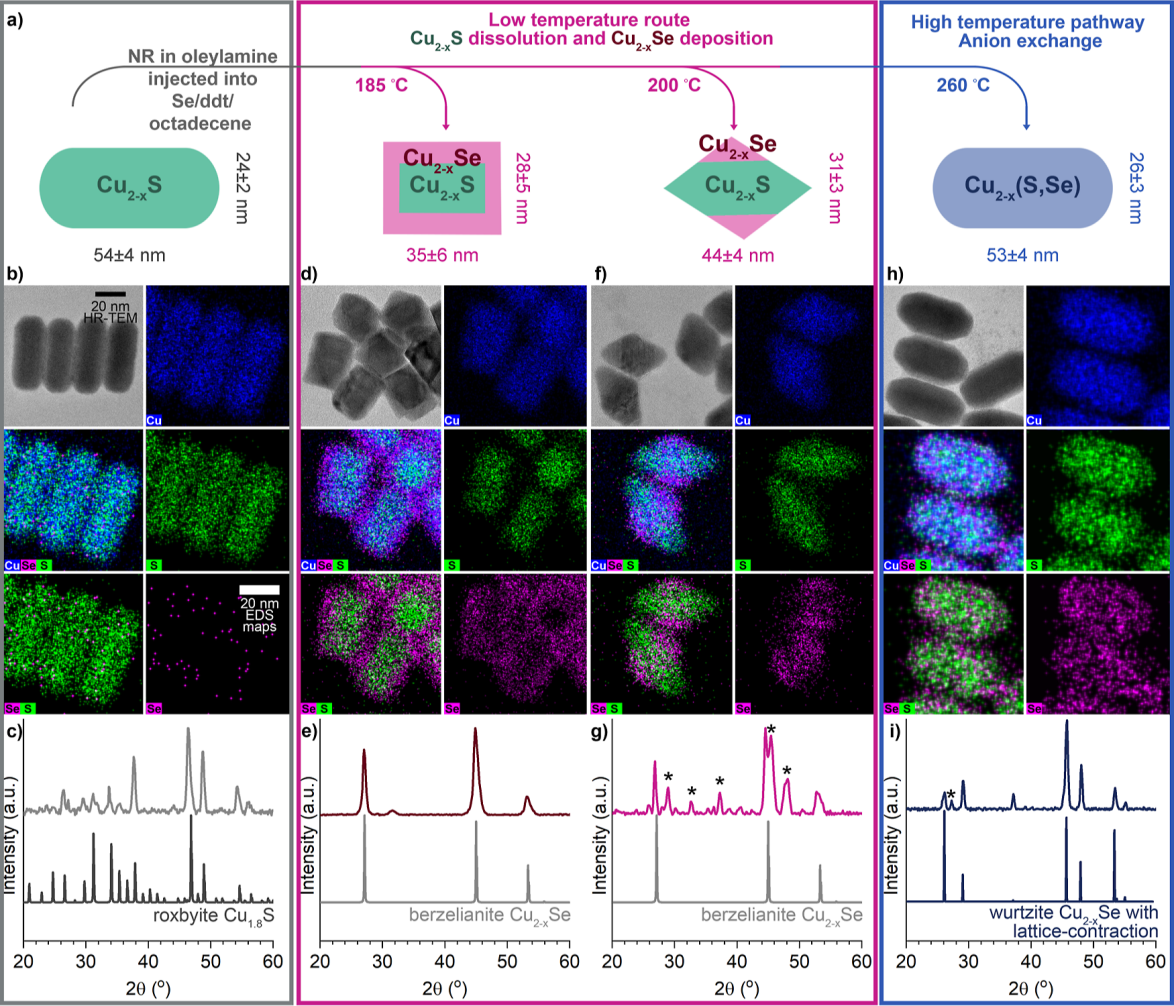
(a) Schematic representation of the process of postsynthetic transformation of Cu_2–*x*_S nanorods into three different Cu_2–*x*_S–Cu_2–*x*_Se nanoheterostructures [Cu_2–*x*_S = teal, Cu_2–*x*_Se = pink, and Cu_2–*x*_(S,Se) = purple] with the particle dimensions. Cu_2–*x*_S–Cu_2–*x*_Se nanoheterostructures resulting from injection of Cu_2–*x*_S nanorods (b,c) into Se/ddt/octadecene mixtures held at either 185 °C (d,e), 200 °C (f,g), or 260 °C (h,i) for 2 h. HR-TEM shows changes in particle morphology at lower temperatures from rods (b), to faceted bricks (d), and to rhombuses (f), while at high temperature, (h) the rod morphology is maintained. STEM-EDS maps (blue = Cu, green = S, and magenta = Se) show how the integration of Cu_2–*x*_Se changes. A Cu_2–*x*_S/Cu_2–*x*_Se core/shell is formed at 185 °C (d). Triangle-shaped deposits of Cu_2–*x*_Se around a faceted primarily Cu_2–*x*_S core form at 200 °C (f). S and Se are evenly distributed with Cu at 260 °C (h). PXRD demonstrates an evolution in crystal structure from the initial roxbyite Cu_1.8_S structure (c, compared to ICSD 00-023-0958), to cubic berzelianite Cu_2–*x*_Se at 185 °C (e, compared to ICSD 01-088-2043), to berzelianite with a secondary phase (g), and to lattice-contracted wurtzite^31^ at 260 °C (i).

Injection of roxbyite-phase Cu_2–*x*_S nanorods suspended in oleylamine into a Se/ddt/octadecene mixture (see Experimental Section) at three different temperatures, 185, 200, and 260 °C, resulted in a different Cu_2–*x*_S/Cu_2–*x*_Se nanoheterostructure at each temperature, as shown schematically in Figure 1a. These nanoheterostructures differ in shape and crystalline phase as well as extent and regioselectivity of Se incorporation, as discussed in detail below. Figure 1 shows Cu_2–*x*_S nanorods after 2 h of transformation at three different injection temperatures. Injection at 185 °C produces a Cu_2–*x*_S/Cu_2–*x*_Se core/shell nanobrick (Figure 1d,e). Injection at 200 °C produces a Cu_2–*x*_S/Cu_2–*x*_Se core/shell nanorhombus (Figure 1f,g). Injection at 260 °C produced a Cu_2–*x*_(S,Se) nanorod (Figure 1h,i).

After heating Cu_2–*x*_S rods in the Se/ddt/octadecene reaction mixture at 185 °C (the lowest of the chosen temperatures) for 2 h, the nanorods (Figure 1b) have transformed into brick-like shapes (Figure 1d), but the population was not homogeneous (Figure S1). A Cu_2–*x*_Se shell forms uniformly on the edges (Figure 1d). The particles reproducibly became shorter and wider, acquiring additional facets. The rods were initially 54 ± 4 × 24 ± 2 nm; the length shrank to 35 ± 6 nm and the width expanded slightly to 28 ± 5 nm (Figure S1). Lattice fringes appear in the center of the particle, where S is concentrated, indicating some crystallinity in this region. The PXRD (Figure 1e), however, shows only cubic Cu_2–*x*_Se. This suggests the disruption of the crystal phase in the Cu_2–*x*_S core and a crystalline Cu_2–*x*_Se shell. Crystallization of the cubic Cu_2–*x*_Se phase on the exterior of the particle may encourage a phase change in Cu_2–*x*_S to minimize interfacial strain.

After heating Cu_2–*x*_S rods in the Se/ddt/octadecene reaction mixture at 200 °C (the intermediate chosen temperature) for 2 h, the particles have a rhombus shape. STEM-EDS maps show a faceted rod core of primarily Cu_2–*x*_S with triangular deposits of Cu_2–*x*_Se on opposite sides to create a rhombus shape. Similar to the nanobricks, the particles are shorter (44 ± 4 nm long) and wider (31 ± 3 nm wide) than the original nanorods (Figure S1). The rhombus shape is formed at 200 °C more consistently than the brick population at 185 °C. PXRD (Figure 1g) shows both cubic Cu_2–*x*_Se and an additional pattern that might indicate initial formation of a solid solution and is discussed in more detail below. Lattice fringes extend across the Cu_2–*x*_S core into the Cu_2–*x*_Se deposits, indicating epitaxial growth of Cu_2–*x*_Se (Figure 1f).

After heating Cu_2–*x*_S rods in the Se/ddt reaction mixture at 260 °C (the highest temperature chosen) for 2 h, the particles retain the rod shape and homogeneously incorporate Se throughout the particle (Figure 1h). The rods become slightly more faceted but overall maintain morphology in a way that suggests Se is incorporated through an anion-exchange process (53 ± 4 × 26 ± 3 nm, statistically indistinguishable from the original Cu_2–*x*_S rods) (Figure S1). The PXRD pattern (Figure 1i) matches that of wurtzite copper selenide but with all five major peaks shifted to higher 2θ values.^31,57^ The peaks at 47.3 and 44.8° 2θ in the reported wurtzite pattern shift to 48.0 and 45.7° 2θ in the experimental pattern, respectively. The shift in the experimental pattern to 2θ values higher than the wurtzite reference indicates that the close-packed anion planes are closer together due to lattice contraction to accommodate smaller S^2–^ ions in a Cu_2–*x*_(S,Se) solid solution. Such a solid solution is consistent with the homogeneous S and Se distributions (Figure 1h).

Why is it that injecting the same nanoparticles into the same reaction mixture for the same amount of time but with different temperatures should result in such different particles? At these three different temperatures, we observe different shapes, different regioselectivities, and different crystal structures: 185 °C affords core/shell Cu_2–*x*_S/Cu_2–*x*_Se nanobricks with a cubic Cu_2–*x*_Se structure, 200 °C affords core/shell Cu_2–*x*_S/Cu_2–*x*_Se nanorhombuses with a cubic Cu_2–*x*_Se structure, and 260 °C affords alloyed Cu_2–*x*_(S,Se) nanorods at 260 °C with a hexagonal Cu_2–*x*_Se crystal structure. One hypothesis is that there is a continuous evolutionary pathway, and we just happen to have sampled three distinct points along that pathway. This would indicate that we could choose one temperature and obtain the three different outcomes by selecting an early time (to obtain the core–shell bricks) or later time (to obtain the alloyed rods). A counter hypothesis is that there are distinct mechanisms driving the formation of each of the three particle types. If this were the case, then we would observe three distinct pathways over time. To identify and explain the origin of these different Cu_2–*x*_S–Cu_2–*x*_Se-containing nanoparticles, we examined the evolution of particles over time at each of the three key reaction temperatures, 185, 200, and 260 °C. As discussed in detail below, we posit that there are two transformation pathways in competition. A high-temperature transformation pathway occurs above 200 °C and results in the integration of Se into the rods through anion exchange. The lower-temperature transformation pathway occurs at 185 and 200 °C and involves coincident Cu_2–*x*_S dissolution and Cu_2–*x*_Se deposition and shape change (Figure 1a).

### High-Temperature Transformation Pathway—Cu_2–*x*_(S,Se) Alloy Formation through Anion Exchange

**Figure 2:**
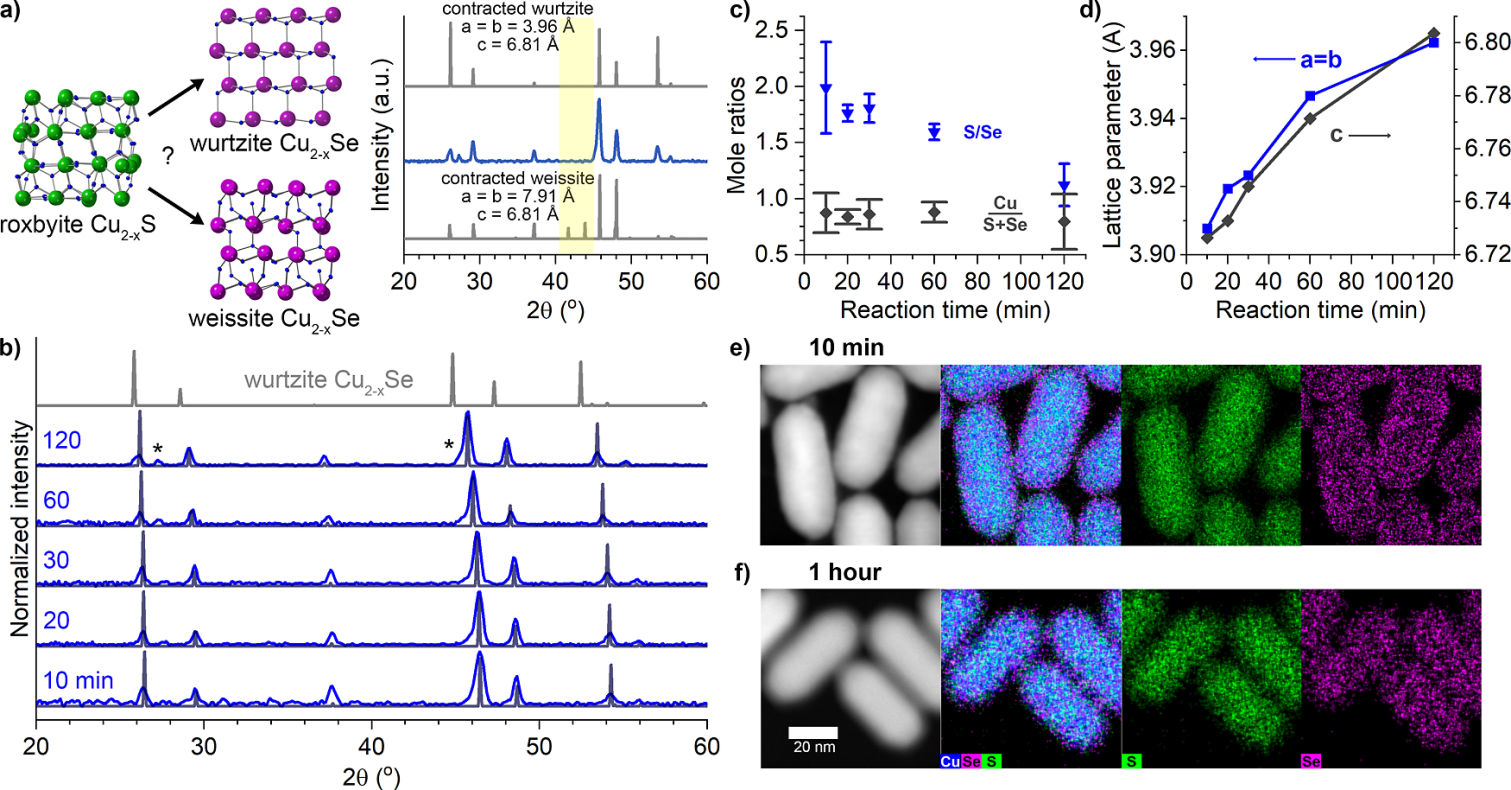
Cu_2–*x*_S nanorods reacted in Se/ddt at 260 °C to form a solid solution of Cu_2–*x*_(S,Se) with reaction times varying from 10 to 120 min. (a) Comparison of the crystal structures and PXRD patterns of the roxbyite Cu_2–*x*_S starting phase and two possible Cu_2–*x*_Se phases with the hexagonally close-packed anion sublattice, wurtzite and weissite. (b) Experimental PXRD patterns of particles at different reaction times. The overlaid patterns show the wurtzite Cu_2–*x*_Se pattern^31^ (top), and wurtzite patterns matched to the experimental patterns by varying the lattice parameters as shown in (d). Asterisks indicate a small berzelianite impurity phase. (c) Mole ratios measured by SEM-EDS over reaction time, showing that the cation/anion mole ratio remains constant but that the S/Se ratio decreases over time as Se replaces S. (d) Simulated wurtzite lattice parameters over reaction time showing a steady lattice expansion with respect to the original roxbyite particles that stops short of the pure Cu_2_Se end-member parameters (*a* = *b* = 4.04 Å and *c* = 6.89 Å). (d,e) STEM-EDS maps of particles after 10 min (e) and 1 h (f) of the reaction showing that the homogeneous distribution of S and Se persists across all tested times.

The progression of Cu_2–*x*_S nanorods after exposure to the Se/ddt reaction mixture at 260 °C, the highest temperature examined, was monitored by aliquots removed at 10, 20, 30, 60, and 120 min (Figure 2b–f). During ion exchange, a newly introduced element replaces an existing element, while particle shape and aspects of the initial crystal structure are typically^58,59^ retained. It is possible for anion layer shifts to create stacking faults and phase conversion.^4,60^ The consistency of the shape and crystal structure showed that Se was incorporated through an anion exchange. Particles transformed at 260 °C for various times show a continuous variation in the S/Se ratio while maintaining a constant cation/anion ratio and homogeneous elemental distribution, supporting the fact that anion exchange is producing a Cu_2–*x*_(S,Se) alloy. The steady decrease in the S/Se mol ratio over time as the particles are transformed at 260 °C (Figure 2c) while the Cu/anion ratio remains unchanged is consistent with the replacement of S^2–^ ions by Se^2–^ ions.

Transformation of the pseudohexagonal roxbyite phase of Cu_2–*x*_S to a metastable hexagonally close-packed phase indicates retention of the anion sublattice typical of ion exchange. The unreacted Cu_2–*x*_S rods match the Cu_1.8_S roxbyite phase (ICSD 00-023-0958, Figure 1c) which has a distorted hexagonally close-packed S^2–^ sublattice. Cu^+^-rich layers of trigonally coordinated Cu^+^ ions alternate with sparser layers of three- and fourfold coordinated Cu^+^.^61^ At the 10 min reaction time, the most prominent PXRD peaks match those of the recently reported wurtzite phase of Cu_2–*x*_Se (Figure 2a,b, top)^31^ with lattice plane contraction due to the presence of both S and Se. There remain indicative of the roxbyite phase that disappears into the background noise at later times. The overlaid patterns in Figure 2b and lattice parameters reported in Figure 2d were obtained by changing the lattice parameters of the wurtzite Cu_2–*x*_Se pattern in CrystalDiffract to match the observed pattern. Samples transformed at 260 °C between 10 min and 2 h show a continual shift in the major diffraction peaks to lower 2θ (Figure 2b). This is consistent with the expansion of the crystal lattice due to the incorporation of Se and the formation of a wurtzite Cu_2–*x*_(S,Se) solid solution. While the PXRD peaks are a close match to the wurtzite structure, there are other hexagonal polytypes of Cu_2–*x*_Se and the possibility of stacking faults to consider with the structure. Figure 2a compares the crystal structure of roxbyite copper sulfide to that of wurtzite and weissite copper selenide,^30^ aligned with the close-packed anion layers perpendicular to the length of the rod as observed from HR-TEM.^62^ The wurtzite phase contains uniform layers of Cu^+^ ions in a trigonal coordination. The weissite structure, however, is more similar to roxbyite. Weissite exhibits the same alternating Cu^+^-rich and Cu^+^-poor layers with a mixture of trigonally and tetrahedrally coordinated ions as does roxbyite. Comparison of the PXRD patterns of the wurtzite and weissite structures shows that they differ only in two small peaks between 40 and 45° 2θ, highlighted in yellow. As observed in Te^2–^ exchange on weissite Cu_2–*x*_Se,^42^ these small peaks could be suppressed by cation disorder induced by ion exchange. Thus, it seems likely that a disordered phase of weissite, indistinguishable from wurtzite by PXRD, could be forming here.

There was a small amount of cubic close-packed berzelianite that appeared and shifted as the reaction time increased (Figures 2b and S2). The small peak at 27.7° 2θ in the 20 min sample shifted to 27.4° 2θ by 120 min. The low-2θ shoulder of the ∼46° 2θ peak is consistent with the major diffraction peak for berzelianite. This could be a berzelianite impurity phase that also incorporated Se through the course of the anion-exchange reaction, although high-angle annular dark-field (HAADF) images do not show deposits. This would suggest that a small amount of Cu_2–*x*_Se deposition is occurring at 260 °C and indicate that the two pathways describe dominant behaviors, not an exclusive process. Alternatively, stacking faults may introduce a small amount of this cubic phase but this typically introduces uneven edges that are not observed in the HAADF images.^4,41,63^

### How Does This Compare to Te^2–^Anion Exchange?

This discovery of conditions to carry out Se^2–^ exchange on Cu_2–*x*_S follows a recent report of Te^2–^ exchange on the same starting materials.^41^ The driving force for Te^2–^ exchange was a replacement of Te in Te = trioctylphosphine. Simply replacing Te with Se in this reaction did not result in Se exchange; thus, we replaced TOP with ddt. Notably, the Se^2–^ exchange in ddt proceeds without the formation of Kirkendall voids, similar to the Te^2–^-exchange behavior. The STEM-HAADF images (Figures 1h and 2e,f) show that the rod morphology of the Cu_2–*x*_S starting materials is retained across all times evaluated. Given that the ion mobility of incoming and outgoing ions is balanced for Te^2–^ and S^2–^ ions, the more similarly sized Se^2–^ and S^2–^ should also be sufficiently balanced to not cause void formation. Three notable differences in behavior between the Te^2–^exchange in TOP and the Se^2–^ exchange in ddt are observed. First, the Te^2–^ exchange proceeds through three different core-/shell-type regioselectivities before full conversion, while Se^2–^ exchange on the same Cu_2–*x*_S rods forms a solid solution. The various regioselectivities that can result from partial cation exchange can be categorized by the miscibility of phases.^64^ The anion crystal radius is much more similar between S^2–^ (1.84 Å) and Se^2–^ (1.98 Å) than between S^2–^ and Te^2–^ (2.21 Å),^65^ reducing lattice strain and promoting formation of a Cu_2–*x*_(S,Se) solid solution that avoids the interfacial energy due to lattice mismatch. A second notable difference between the prior Te^2–^ anion exchange of roxbyite nanorods and the Se^2–^ anion exchange observed here is the change in the cation/anion ratio. Both Se^2––^ and Te^2–^ exchanges increase the copper deficiency of the resultant particles compared to the initial Cu_2–*x*_S rods, presumably because Cu^+^ vacancies can help accommodate the movement of large anions through the crystal lattice. For Te^2–^, a continual removal of Cu^+^ ions is observed that would help accommodate the Te^2–^ ions. For Se^2–^ exchange, the cation/anion ratio does not increase further as more Se^2–^ replaces S^2–^ (Figure 2c). Last, Te^2–^ anion exchange^31^ unambiguously formed the weissite structure without the cation disorder seen in the Se^2–^ exchange.

### Low-Temperature Transformation Pathway—Cu_2–*x*_Se Deposition and Shape Change

After 2 h of reaction in the Se/ddt reaction mixture at 185 and 200 °C, Cu_2–*x*_S rods formed Cu_2–*x*_S/Cu_2–*x*_Se core/shell nanoheterostructures with dramatically different particle morphologies at each temperature (Figure 1d–g). To better understand this transformation, the phase, morphology, and composition were monitored using aliquots at 10, 20, 30, 60, and 120 min at each temperature. These data were used to determine that Cu_2–*x*_Se originated in a deposition process, rather than anion exchange as was observed at high temperature. The change in particle shape over time, temperature, and the presence of Se was used to identify the conditions for shape change.

### Evidence for Cu_2–*x*_Se Deposition

**Figure 3:**
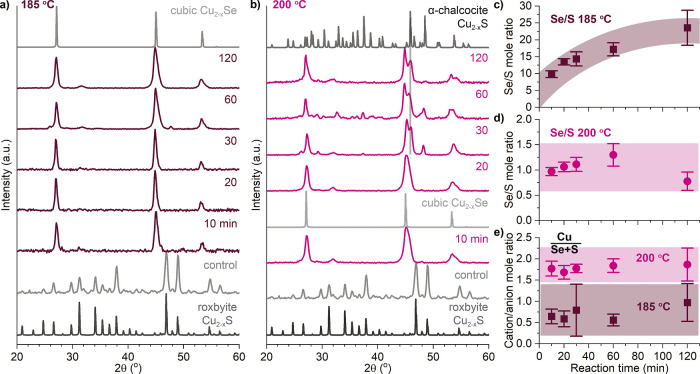
Cu_2–*x*_S nanorods reacted in Se/ddt at 185 and 200 °C to form structures with Cu_2–*x*_S cores and deposited Cu_2–*x*_Se. PXRD patterns of the samples reacted at 185 (a) and 200 °C (b) show that roxbyite copper sulfide (ICSD 00-023-0958) converts to primarily cubic copper selenide (ICSD 01-088-2043), with a new phase emerging at longer reaction times at 200 °C that is similar to α-chalcocite (ICSD 00-023-0961). EDS gives the S/Se mol ratios [(c) 185 and (d) 200 °C] and Cu/(S + Se) ratios (e).

**Scheme 1:**
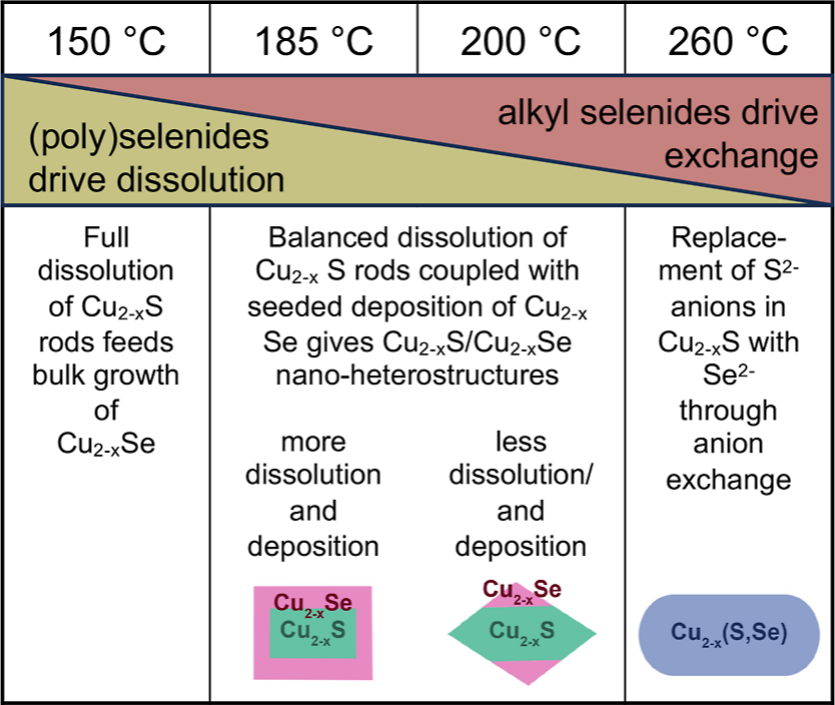
Representation of the Proposed Overall Mechanism Where the Dominant Selenide Species in Solution Varies with Temperature to Drive the Observed Selenization of Cu_2–*x*_S Nanorods

At 185 and 200 °C, Cu_2–*x*_Se is likely formed by seeded deposition as Se species in solution react with Cu^+^ ions released by the gradual dissolution of Cu_2–*x*_S with greater Cu_2–*x*_Se formation at lower temperatures. The possibility of anion exchange can be ruled out by the dramatic changes to the morphology compared to the original rods (Figure 1b,d,f). Further evidence comes from the formation of the more thermodynamically favorable cubic close-packed copper selenide phase known as berzelianite rather than the hexagonally close-packed phase that would be typical of an exchange process. After just 10 min of reaction at the lower temperature (Figure 3a), the only crystalline phase apparent in the PXRD pattern is that of the thermodynamically most stable cubic copper selenide, with three major peaks at 27.1, 44.9, and 53.3° 2θ that correspond to the pattern generated from ICSD 01-088-2043 with no trace of the original roxbyite Cu_1.8_S phase. This cubic phase dominates the crystal structure at 185 °C for the whole 2 h period examined, despite the continued existence of a copper sulfide domain apparent in the STEM-EDS maps that exhibit lattice fringes in the HRTEM images. EDS (Figure 3c,e) shows a large amount of Se at 10 min (10 Se/S mole ratio) that roughly doubles by 2 h. The cation-to-anion mole ratio drops significantly for the particles reacted at 185 °C but stays consistent across time. This is consistent with dissolution of Cu_2–*x*_S to supply the Cu^+^ necessary to react with Se^2–^ in solution to form cubic Cu_2–*x*_Se. At 200 °C (Figure 3b), berzelianite is the only phase present at lower reaction times, but a new phase emerges at 30 min with peaks at 45.4 and 47.9° 2θ as well as several small peaks at 30–45° 2θ. These match quite well to the α-chalcocite copper sulfide phase and might be due to recrystallization within the copper sulfide core. This might also indicate the very beginnings of a wurtzite-like solid solution (Figure S3), promoting the idea that the two mechanistic pathways are not completely exclusive but have dominant behaviors along a continuum. Lower temperatures apparently promote greater dissolution. Much less Se is incorporated at 200 °C (Figure 3d), staying at about 1 Se/S mole ratio across different times, whereas the Se/S ratio is more than 20 after 2 h of reaction at 185 °C (Figure 3c). Furthermore, the cation/anion mole ratio drops to ∼1 at 185 °C, indicating a very large number of copper vacancies, while at 200 °C, the cation/anion ration remains close to the starting material. Both of these observations can be explained if there is greater dissolution of Cu_2–*x*_S feeding more, faster growth of Cu_2–*x*_Se at 185 °C resulting in more Cu_2–*x*_Se with greater copper deficiency at 185 °C than at 200 °C. Further evidence is seen in the particle sizes. The original Cu_2–*x*_S particles are 54 ± 4 nm long; the particle length drops to 44 ± 4 nm at 200 °C and all the way to 36 ± 6 nm at 185 °C (Figure S1). Typically, lower temperatures slow reactions like dissolution; observing greater dissolution and accompanying Cu_2–*x*_Se deposition at lower temperatures are unusual features of this system. Rationalizing this is a key component of the overall mechanism discussed later (Scheme 1).

### Evidence That Cu_2–*x*_Se Deposition and Shape Change Are Separate Processes

**Figure 4:**
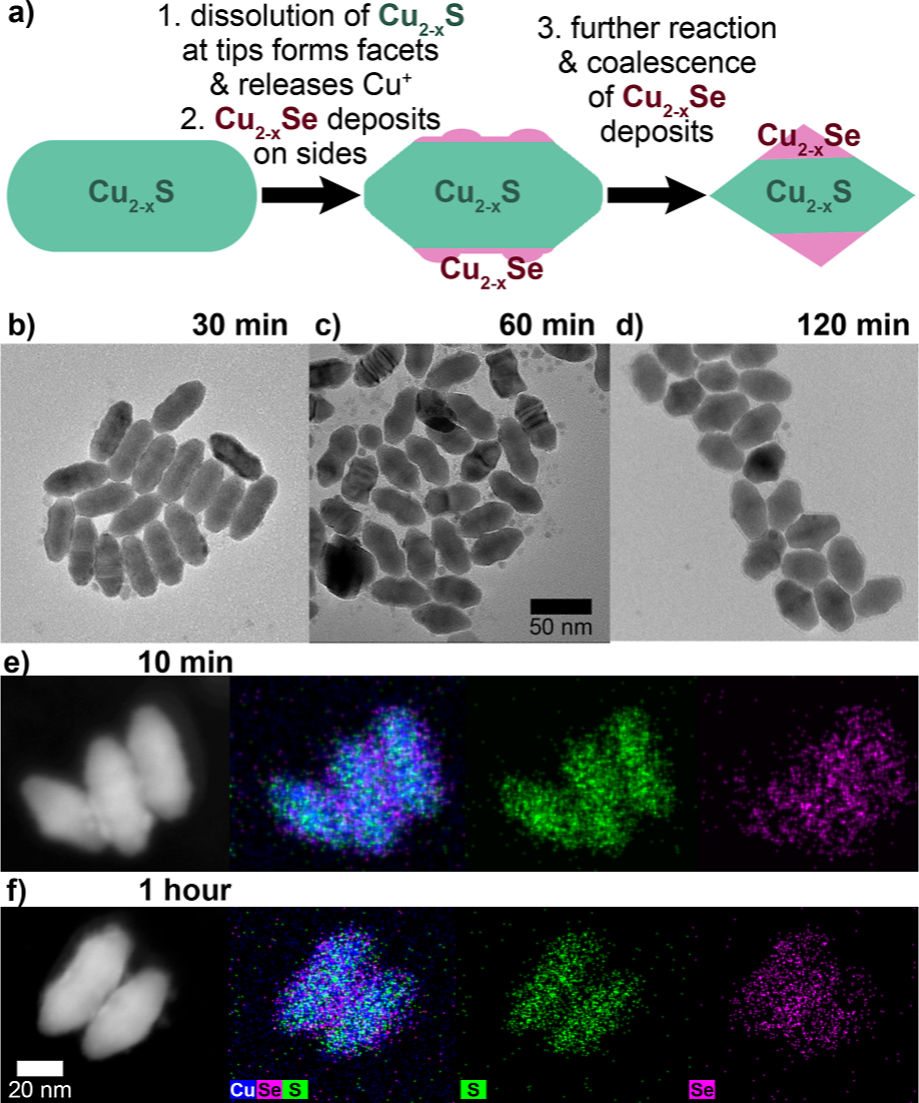
(a) Schematic representation of the conversion of Cu_2–*x*_S nanorods to Cu_2–*x*_S–Cu_2–*x*_Se nanorhombuses by concerted Cu_2–*x*_S dissolution and Cu_2–*x*_Se precipitation. TEM (a–c) of Cu_2–*x*_S nanorods reacted in Se/ddt/octadecene at 200 °C for 30 min, 1 h, and 2 h shows the shape evolution as the tips sharpen and deposits form on the sides of the rods. STEM-EDS at 10 min (e) and 1 h (f) shows that the deposits are Cu_2–*x*_Se confined to the edges and start to coalesce at 1 h.

Exposure of roxbyite nanorods to Se/ddt at 185 and 200 °C results in different shapes at the different temperatures after 2 h of exposure (Figure 1). This raises questions about the evolution of these shapes and what is causing the shape change. First, control experiments were carried out using the same procedure as the Se/ddt transformation but without Se. ddt and octadecene were heated to either 185 or 200 °C for 2 h, and then particles were injected in oleylamine as usual (Figure S4) rods transformed to spheres with a uniform population. Similar behavior has just recently been reported where ddt causes transformation to spheres and *t*-ddt causes a variety of shapes, all with the same volume as the initial Cu_2–*x*_S nanorods.^45^ The vacancies that are present and increased by interaction with thiols help promote reshaping of the lowest-surface area volume. Other reports show that ddt promotes shape transformation,^33^ multiple surface-binding modes,^44^ vacancy formation, and particle self-assembly.^43^ Examining the shapes over time and temperature reveals quite complex behavior (Figures 4b and S6). At 200 °C (Figure 4b), we monitored the shape evolution as the tips of rods begin to sharpen as Cu_2–*x*_S is etched away at 30 min, and a new facet is exposed suggesting that this surface is stabilized by interaction with a specific solution species. Simultaneously, Cu_2–*x*_Se begins to form small deposits on the sides of the rods by 10 min as shown in STEM-EDS (Figure 4e) that start to coalesce by 1 h (Figure 4f). As the reaction progresses to 1 and 2 h (Figure 4c,d), the faceting continues to sharpen the tips of the underlying Cu_2–*x*_S rod and the deposited Cu_2–*x*_Se coalesces into a triangular pattern to give the nanorhombus shape at 2 h. This overall process is schematically shown in Figure 4a. Cu_2_S (Δ*H* = −79.5 kJ/mol) is more thermodynamically stable than Cu_2_Se (Δ*H* = −39.5 kJ/mol),^66^ and therefore the behavior where Cu_2_S dissolves while Cu_2_Se deposits must be a kinetically driven behavior. The large concentration of selenides in solution is likely driving dissolution of both Cu_2_S and Cu_2_Se while promoting equilibrium with redeposition of primarily Cu_2_Se. At 185 °C (Figure S5), early times show formation of spheres that evolve into several different faceted shapes, again suggesting an interaction between specific surfaces and solution species that guide shape change. Small particles are observed after heating with Se at 185 °C but not in the control samples. Such deposition further indicates that Cu^+^ ions are being dissolved at sufficient rates to allow formation of very small particles, even though the chemistry of these tiny particles was not measurable with STEM-EDS. Reactions at an even lower temperature of 150 °C resulted in growth of very large particles of cubic Cu_2–*x*_Se in triangles and hexagons with diameters on the order of ∼150 nm, in comparison to the 50 nm long rods (Figure S6). This suggests that growth of Cu_2–*x*_Se is generally preferred under lower-temperature conditions, and the 185 and 200 °C range where deposition is controlled is a transition point in a larger spectrum of behaviors.

### Why Are There Two Different Postsynthetic Transformation Routes?

Uncovering why two distinct temperature-dependent transformation pathways are observed is complicated because the two transformation pathways are different in several important respects. Why is it that the lower-temperature pathway results in significant shape change and evolution of the shape over time, while the higher-temperature pathway maintains the rod morphology? Why is it that the lower-temperature pathway maintains phase segregation of the Cu_2–*x*_S and cubic Cu_2–*x*_Se components, while the higher-temperature pathway forms an alloy of wurtzite Cu_2–*x*_(S,Se)? Why does the lower-temperature pathway proceed through deposition, while anion exchange occurs at higher temperatures? We posited that evolution of the Se-containing solution species with temperature affects both the surface chemistry, phase, and rapidity of Cu_2–*x*_S dissolution and Cu_2–*x*_Se growth and is key to answering these questions. Supporting this idea is the observation that the color of the Se/ddt/octadecene solution before nanorod injection changes as the temperature increases. The solution color evolves from clear yellow at 160 °C, to orange-gold at 190 °C, then to gold at 210 °C, and finally to darker yellow at 260 °C. This color change indicates a significant alteration of the Se solution chemistry in which the transformations take place; similar changes do not occur when heating ddt or octadecene alone.

### Role of Surface Chemistry

**Figure 5:**
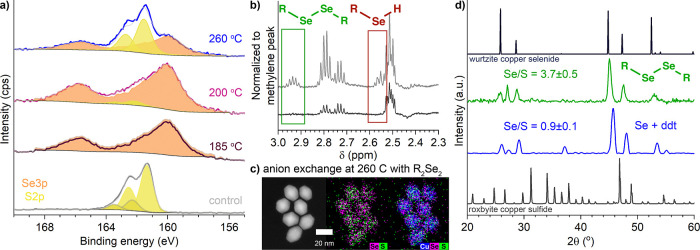
Characterization of reaction solution and surface species through (a) XPS of particles transformed at 185, 200, and 260 °C and (b) ^1^H NMR of the ddt/octadecance solution heated to 260 °C with (top) and without (bottom) Se, showing evidence for formation of didodecyl diselenide and dodecylselenol. Cu_2–*x*_S nanorods transformed by reaction with didecyl diselenide in place of Se/ddt at 260 °C characterized by (c) PXRD and (d) STEM-EDS.

To better understand how the evolving solution chemistry might be altering the surface chemistry of the particles, we compared XPS (Figures 5a and S7) on particles transformed at 185, 200, and 260 °C with control samples of Cu_2–*x*_S nanorods. XPS of particles transformed using the low- and high-temperature pathways shows a large difference in the amount of Se at the surface due to both the chemistry of the particle and the identity of the surface ligand. The S 2p/Se 3p region for the original Cu_2–*x*_S nanorods shows substantial amounts of sulfur due to both the thiol ligands (at higher binding energy) and the sulfur at the surface of the particle (at lower binding energy), producing two pairs of S 2p peaks as previously reported for surface-bound dodecanethiol-capped copper sulfide.^44,67^ The particles transformed with the lower-temperature pathway (185 and 200 °C) show large Se 3p peaks but not a convincing trace of sulfur. Instead of the thiol-based ligand commonly observed for reactions occurring in ddt,^44^ the surface is terminated by a Se species. There is a Se signal due to the outer layer of Cu_2–*x*_Se on the particle, but the lack of a S signal suggests that a Se-containing surface ligand is in place. The absence of S and N (which could original from an oleylamine ligand) signals and the large excess of Se (Se/S = 7.3) further indicate that a selenium-containing ligand is terminating the surface. From this we infer the presence of a Se-containing solution species that binds strongly to the particle surface. As the reaction temperature increases to 260 °C, the S 2p signal returns on top of a Se 3p signal. This can reflect the homogeneous mixture of both Cu_2–*x*_S and Cu_2–*x*_Se that makes up the alloy and leaves open the possibility that either a sulfur- or selenium-containing surface ligand is present. Examination of the Se 3d region looks nearly identical for particles transformed at 185, 200, and 260 °C. Two sets of Se peaks are present, further supporting that the particles have surface selenium- and selenium-containing ligands. This investigation of the surface chemistry revealed that at 185–200 °C, the solution chemistry must be dominated by a species that promotes Cu_2–*x*_Se growth and largely displaces the thiol ligands. Selenide ions play both of these roles, serving as ligands on metal sulfides^68^ and driving their formation.^47,69^

### Role of Solution Chemistry

To identify the cause of the color change of the ddt/octadecene/Se reaction mixture and identify species that might form a Se-containing surface ligand, we heated the components from the reaction mixtures without nanorod injection and examined the solutions with ^1^H NMR (Figures 5b and S8). ^1^H NMR of ddt heated with or without Se showed that the solution species did not change significantly with temperature or the presence of Se (Figure S8), only showing formation of the disulfide. Polyselenide species could reasonably form under these conditions, similar to the formation of polysulfides when sulfur is heated in ddt.^28^ These would cause the observed yellow color^70^ without altering the ^1^H NMR. ddt heated with octadecene with or without Se showed significant differences between 185 and 260 °C. At the lower temperature, no new peaks were apparent that would suggest an alkyl-Se species. At 260 °C with Se, peaks indicative of both dialkyl diselenide and alkyl selenol appear that match those of didodecyl diselenide and dodecyl selenol.^31^ The reduction of the double bond in octadecene seems to play a crucial role in reducing elemental Se and forming Se–C bonds. The double bond in octadecene can produce a polymer impurity^48^ and can reduce elemental selenium to a variety of species including H_2_Se^49^ and polymeric selenium species.^71^ Ho et al. have shown that dodecyl selenol can react with octadecene and oleylamine to form didodecyl diselenide and didodecyl selenide at 220 °C but not at 155 °C.^52^

### Impact of Solution Species on Crystal Phase of Cu_2–*x*_Se

Based on the XPS and ^1^H NMR evidence suggesting the presence of polyselenides at lower temperatures and alkyl selenides at higher temperature, we reviewed the literature on the effect of reactive Se compounds on copper selenide growth to put the behavior of these species into context. Diorganyl dichalcogenides undergo different thermal decomposition routes depending on solvent^72^ and have been used to target metastable semiconductor nanocrystal phases.^51^ Hernández-Pagán et al.^31^ developed a phase-selective synthesis of wurtzite Cu_2–*x*_Se nanoplatelets, where the use of didodecyl diselenide as the selenium source produced wurtzite phase, while Lord et al.^30^ used diphenyl diselenide to produce a metastable weissite structure. Similarly, the metastable wurtzite phase of Cu_2–*x*_Se can be produced by the reaction of dodecyl selenol with octadecene to yield selenide or diselenide at 220 °C or through ligation effects with long-chain amine at 220 or 155 °C.^52^ Dodecyl selenol, on the other hand, reacts directly to form Cu_2–*x*_Se in either thermodynamically preferred cubic phase^31^ or under slightly different conditions, the umangite phase.^32^ They attribute this behavior to the fact that dodecyl selenol forms a reactive Cu–selenoate complex that readily nucleates into Cu_2–*x*_Se, whereas the Se–Se bond in dodecyl diselenide prevents formation of such a complex and instead Se slowly combines with Cu^+^ at the particle surface directing formation of the metastable phase. At lower temperatures where we saw no evidence of any alkyl–Se bond formation, it is plausible that the reaction mixture used here with Se, ddt, and octadecene with oleylamine injected along with the nanorods can form (poly)selenides. Combination of Se and oleylamine (with^73^ or without ddt^74^) results in alkylammonium selenides (OLA)_m_Se_n_. Thus, we formed a hypothesis that formation of the three different Cu_2–*x*_S–Cu_2–*x*_Se nanoheterostructures at three different temperatures could be rationalized based on solution chemistry alterations where (poly)selenides dominate at lower temperatures and alkyl selenides dominate at higher temperatures, with dialkyl diselenides in particular promoting anion exchange.

### Testing the Role of Dialkyl Diselenides on High-Temperature Transformation

Two further studies were performed to confirm that alkyl selenide formation is key to the anion exchange observed at higher temperatures. First, we altered the source of the alkyl chain, the thiol solvent. Replacing ddt with either tetradecanethiol or *tert*-ddt reveals that the thiol species is important (Figure S9). Replacing ddt with tetradecanethiol results in cubic Cu_2–*x*_Se at both 200 and 260 °C—no anion exchange occurs at 260 °C. At 200 °C, broad peaks in PXRD show nanocrystalline particles indicative of seeded deposition. At 260 °C, bulk Cu_2–*x*_Se suggests the dissolution of Cu_2–*x*_S nanorods to grow large Cu_2–*x*_Se particles. Replacing ddt with *t*-ddt, however, showed a mixture of deposition and anion exchange at 185 °C. If formation of dialkyl diselenides is an essential step in anion exchange, then it is logical that the identity of the alkyl species would modulate formation and propensity for anion exchange as it can alter thermal decomposition.^72^

Given evidence that dialkyl diselenide forms at high temperature and could promote the formation of the wurtzite Cu_2–*x*_Se phase, didecyl diselenide was synthesized directly and used in place of Se and ddt in a transformation at 260 °C (Figure 5c,d). Didecyl diselenide (0.17 mmol) was added in place of Se (0.30 mmol) and ddt. Validating the supposition that didecyl diselenide directs anion exchange, PXRD showed a lattice-contracted wurtzite Cu_2–*x*_Se and STEM-EDS showed a homogeneous distribution of S and Se. Notably, the shift in the PXRD reflections indicated a greater incorporation of Se compared to the 2 h reaction with Se/ddt. Despite a lower overall amount of Se present in solution, a much larger Se/S mole ratio (3.7 ± 0.5) was observed with didecyl diselenide than with Se/ddt (0.9 ± 0.1), also indicating a greater extent of exchange. Unlike with Se/ddt, the particle shape did change slightly. Rods transformed to a faceted diamond shape that echoes the faceting observed at shorter times for the 200 °C transformation.

### Overall Mechanism

Taking all observations into account, we propose a mechanism where Se, ddt, and octadecene react to form (poly)selenides at relatively low temperatures and alkyl-selenide species at relatively high temperatures and that the balance of these solution species modulates Cu_2–*x*_S dissolution, shape change, Cu_2–*x*_Se growth, and Se^2–^ anion exchange to create the three distinct Cu_2–*x*_S–Cu_2–*x*_Se nanostructures observed at 185, 200, and 260 °C (Scheme 1). At the lowest temperatures (150 °C) at which Se is fully dissolved, rapidly reacting (poly)selenide species bind to Cu^+^ in the nanorods, dissolving the Cu_2–*x*_S nanorods and released Cu^+^ reacts with polyselenides to form large Cu_2–*x*_Se particles (Figure S6). Alkyl ammonium selenides are known to rapidly react with metal species to form metal selenides.^46,47,73^ As the temperature increases to the 185–200 °C range, alkyl-selenide species start to form, reducing the concentration of the (poly)selenides and altering the balance of dissolution of Cu_2–*x*_S and formation of Cu_2–*x*_Se. At 185 °C, this balance still favors Cu_2–*x*_S dissolution and Cu_2–*x*_Se growth, but the dissolution process has slowed enough that deposition is occurring on the remaining Cu_2–*x*_S cores, giving the faceted-brick shape with a thick Cu_2–*x*_Se shell and extensive Se incorporation that increases with reaction time. This coupled dissolution-growth process is supported by the fact that small particles are often observed around the larger particles—there may be some independent formation of Cu_2–*x*_Se clusters. Rapid growth of Cu_2–*x*_Se by the reaction with (poly)selenides would explain why a more thermodynamically favorable cubic phase was observed. At 200 °C, the dissolution of Cu_2–*x*_S is slowed even further as (poly)selenides become alkyl selenides. Dissolution is restricted to the Cu^+^ released as the tips of the rods are becoming faceted. This limited amount of Cu^+^ then forms relatively well-defined Cu_2–*x*_Se domains, specifically on the rod edges. These domains develop into faceted triangles after deposition (Figure 4a). A (poly)selenide species could be the surface ligand at this point, which would explain the XPS that shows primarily Se at the surface, and interactions with (poly)selenide or alkyl selenides could contribute to the observed faceting. The low concentration of solution Cu complex at this point keeps the direct formation of Cu_2–*x*_Se to a minimum and instead promotes Cu_2–*x*_Se formation on the existing Cu_2–*x*_S. Similar deposition of Cu_2–*x*_S onto existing Bi nanoparticles has been reported to vary with the stability of the Cu–thiolate complex, with more stable complexes creating low free Cu-ion concentrations and thus fewer deposition sites.^75^ At 260 °C, the Cu_2–*x*_S dissolution and Cu_2–*x*_Se growth process are outcompeted by reaction with didodecyl diselenide. Didodecyl diselenide reacts at the nanorod surface to provide a Se^2–^ source. This drives anion exchange with a wurtzite structure, as does when Cu_2–*x*_Se is synthesized directly from didodecyl diselenide. The stronger C–Se bonds in alkyl selenides slow the Cu_2–*x*_S dissolution and Cu_2–*x*_Se deposition process compared to (poly)selenides. Alkyl selenide species like dodecyl selenol or dodecyl diselenide could ligate the nanorod surface in an equilibrium with the existing thiol ligands. This will result in a mixture of S and Se on the nanorod surface, as observed in X-ray photoelectron spectra at 260 °C. Such surface ligation could stop the shape change that occurs in ddt alone, maintaining the rod shape when the transformation occurs in Se/ddt. The relatively low concentrations of didodecyl diselenide in the complex Se/ddt reaction mixture could prevent faceting observed when didodecyl diselenide is the only source of Se^2–^. A slow process of exchange would also contribute to balancing the inward and outward mobility of anions. This balanced mobility would not only contribute to the lack of Kirkendall void formation but also could support an equilibrium where Se^2–^ and S^2–^ from the thiol complexes both entered the nanorods.

## Conclusions

Three new Cu_2–*x*_S–Cu_2–*x*_Se nanoheterostructures were formed from reaction conditions that differ only by the temperature. We rationalize these different outcomes from the same Se/ddt/octadecene solution based on the complicated temperature-dependent solution chemistry. At low temperatures, highly reactive solution species (likely polyselenides) promote particle dissolution and limited Cu_2–*x*_Se growth from the freed Cu^+^ ions. At high temperatures, alkyl selenide species including didodecyl diselenide promote slow transformation of Cu_2–*x*_S to an alloy of Cu_2–*x*_(S,Se). The balance of these two species alters with temperature, creating different behavior domains that balance either coupled Cu_2–*x*_S dissolution and Cu_2–*x*_Se deposition or Se^2–^ anion exchange. This work offers new multichalcogenide nanoheterostructures that offer the potential to create even more complex structures through cation exchange and to apply this new chemistry to other metal sulfides. It offers insights into the molecular basis of nanoparticle synthesis and postsynthetic transformations that can inform future rational design of elaborate multicomponent nanostructures.

## Supporting Information Available

Detailed author attributions and additional data (PDF)

## Notes

Raw data files can be accessed through the Open Science Framework at https://osf.io/bk57e/.

## Abbreviations

ddt: dodecanethiol
*t*-ddt: *tert*-dodecanethiol
