# Supplementary material for: Temperature-Dependent Selection of Reaction Pathways, Reactive Species, and Products during Postsynthetic Selenization of Copper Sulfide Nanoparticles
Source: Chem Mater. 2023 Oct 20;35(21):9073–85. doi: 10.1021/acs.chemmater.3c01772 (PMC10653086; doi:10.1021/acs.chemmater.3c01772)
Supplement: Supplementary file 1 — cm3c01772_si_001.pdf [file cm3c01772_si_001.pdf]

Temperature-dependent selection of reaction pathways, reactive species, and products during post-synthetic selenization of copper sulfide nanoparticles

*Brandon Hole,[a ‡] Qi Luo,[a ‡] Ronald Garcia,[a] Wanrui Xie,[a†] Eli Rudman,[a] Chi Loi Thanh Nguyen,[a] Diya Dhakal,[a] Haley L. Young,[b] Katherine L. Thompson,[b] Auston G. Butterfield,[b] Raymond E. Schaak,[c] and Katherine E. Plass[a]**

[a]Department of Chemistry, Franklin & Marshall College, Lancaster, Pennsylvania 17604, United States

[b]Department of Chemistry, The Pennsylvania State University, University Park, Pennsylvania 16802, United States

[c]Department of Chemistry, Department of Chemical Engineering, Materials Research Institute, The Pennsylvania State University, University Park, Pennsylvania 16802, United States

Table of contents	Page
**Table S1.** Author contributions according to CRediT Contribution Roles Taxonomy	**S3**
**Additional Data**	
**Figure S1.** STEM of $Cu_{2-x}S$ nanorods and nanoheterostructures transformed at 185 °C, 200 °C, and 260 °C.	**S4**
**Figure S2.** PXRD of $Cu_{2-x}S$ nanorods transformed at 260 °C.	**S5**
**Discussion: Why only partial exchange?**	**S5**
**Figure S3.** PXRD of $Cu_{2-x}S$ nanorods transformed at 200 °C.	**S6**
**Figure S4.** Effect of injecting $Cu_{2-x}S$ nanorods into ddt and octadecene versus Se, ddt, and octadecene at 185 °C and 200 °C.	**S7**
**Figure S5.** TEM of $Cu_{2-x}S$ nanorods transformed at 185 °C for various times.	**S7**
**Figure S6.** PXRD and TEM of $Cu_{2-x}S$ nanorods in oleylamine injected into Se, ddt, and octadecene at 150 °C and reacted for 2 hours.	**S8**
**Figure S7.** XPS of $Cu_{2-x}S$ nanorods and nanoheterostructures formed by heating for 2 hours at 185 °C, 200 °C, and 260 °C.	**S9**
**Figure S8.** ^{1}H NMR of mixtures of ddt with Se and/or octadecene heated at 185 °C or 260 °C.	**S11**
**Figure S9.** PXRD of $Cu_{2-x}S$ nanorods transformed by exposure to Se and octadecene with various thiols.	**S12**

Table S1. Author contributions according to CRediT Contribution Roles Taxonomy

**Author**	**Contribution**	**Detail**
Brandon Hole	Validation & Investigation, Writing - Review & Editing	Discovered transformation methodology and the three different nanoheterostructures; investigated behavior at 260 °C (Figures 1 and 2)
Qi Luo	Validation & Investigation, Writing - Review & Editing	Investigated behavior at 200 °C at different times (Figures 3 and 4) and at lower temperature (Figure S6) and made XPS samples (Figure S9)
Ronald Garcia	Validation & Investigation, Writing - Review & Editing	Synthesized and investigated the effects of didecyl diselenide (Figure 5) and carried out reactions on reaction mixtures (Figure S8)
Wanrui Xie	Validation & Investigation	Investigated behavior at 185 °C at different times (Figures 3a, S4, S6)
Eli Rudman & Chi Nguyen	Validation & Investigation	Carried out extensive control experiments, made XPS samples (Figure 5) and varied thiol identity (Figure S9)
Diya Dhakal	Validation & Investigation	Modeled crystal structures to match PXRD data (Figure 2)
Haley L. Young, Auston Butterfield, & Raymond E. Schaak	Conceptualization, Methodology, Writing - Review & Editing	Shared unpublished data and ongoing experiments that informed the selection of experiments and interpretation of data for this manuscript
Katherine L. Thompson	Validation	Undertook experiments to determine source of reproducibility issues
Katherine E. Plass	Conceptualization, Methodology, Resources, Writing – Original Draft, Supervision, Project administration, Funding acquisition	Initiated, oversaw, and acquired funding for experiments. Wrote manuscript and created figures.

Additional data

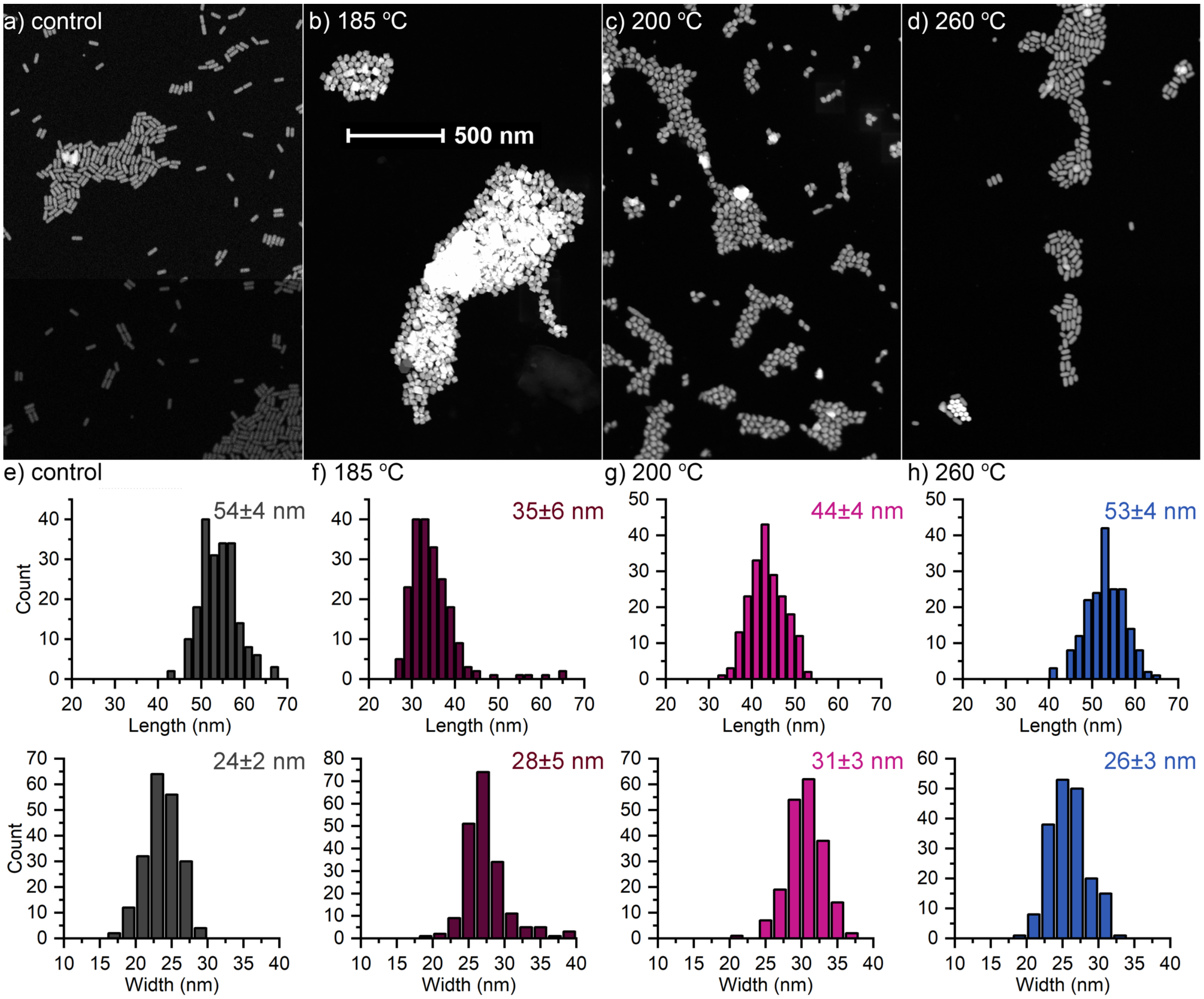

Figure S1. STEM of $Cu_{2-x}S$ nanorods (a) and nanoheterostructures formed by heating for 2 hours at b) 185 °C, c) 200 °C, and d) 260 °C demonstrating the population homogeneity. Particle size measurements of the length and width (e-h) illustrate that particles get shorter and wider at 185 °C and 200 °C, but are unchanged at 260 °C.

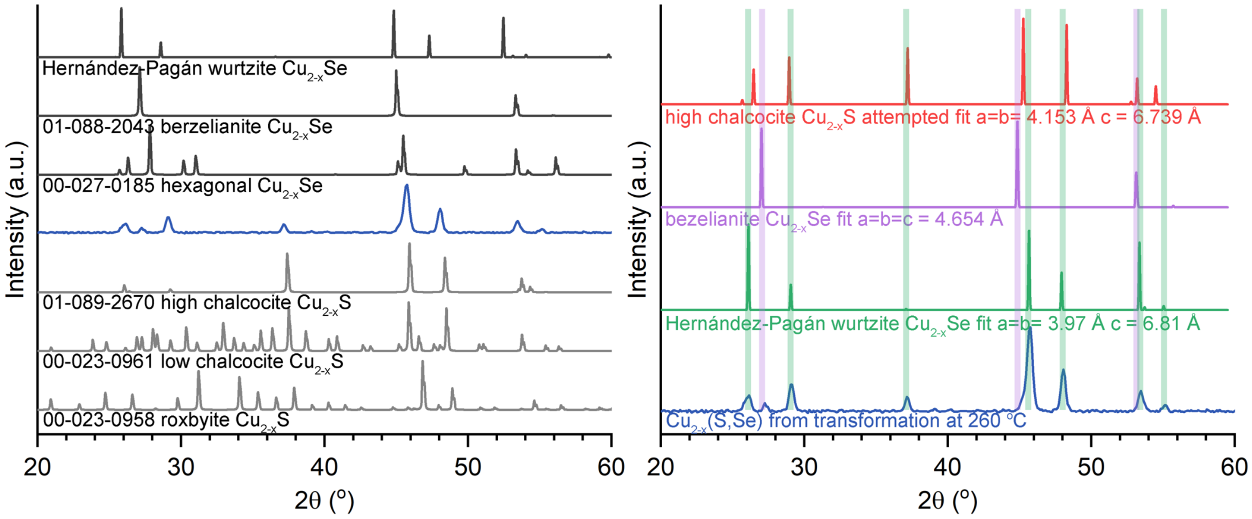

Figure S2. PXRD of $Cu_{2-x}S$ nanorods transformed at 260 °C. a) Comparison with database patterns for various copper sulfide and copper selenide phases, suggesting that it is primarily shifted wurtzite copper selenide. b) A closer comparison of the experimental pattern with cubic berzelianite Cu_2Se, wurtzite Cu_2Se, and high chalcocite Cu_2S suggesting that a small amount of berzelianite is present.

Why only partial exchange? Partial exchange occurs unevenly across reaction times and stops short of full replacement of sulfur by selenium. While the PXRD shift over time indicates lattice expansion due to Se-incorporation over time, the amount of Se incorporated does not increase from 10-30 min and then flattens at a ~1:1 S:Se mole ratio (Figure 2b). From 10 min to 30 min, the most intense (2-10) diffraction peak at ~46 °2θ only shifts by ~0.1 °2θ, while the Se/S mole ratio remains steady. More significant shifts of 0.3 °2θ at 60 min and 0.7 °2θ at 120 min correspond to steady increases in Se/S mole ratio. TEM-EDS mapping of particles at early times appears to have a homogeneous distribution of S and Se similar to later times (Figure 2e,f). This suggests that the solid-solution forms immediately but that the rate at which Se incorporates over time is non-linear. Initially the incorporation is slow with the smaller lattice spacing; it accelerates once the lattice

parameters have expanded sufficiently to more easily allow ion incorporation and mobility. Incorporation of Se would, of course, expand the lattice to allow more Se to follow. Lattice expansion, however, may not be sufficient to encourage further exchange after reaching a 1:1 S:Se mole ratio. Despite a large excess of Se (0.30 mmol of Se to 0.14 mmol of $Cu_{1.8}S$ nanorods), the composition of that alloy does not vary across the whole possible range of compositions (Figure 2c). The S/Se mole ratio only reaches ~1, even when reaction times are extended to 5 hours. The lattice parameters only expand to a=b=3.96 Å and c=6.8 Å, while the pure Cu_2Se wurtzite end-member has a=b=4.04 Å and c=6.89 Å.[31] The presence of sulfur in solution due to the dodecanethiol solvent may provide additional S^{2-} that prevents full conversion to $Cu_{2-x}Se$ or the solution-species driving anion exchange may not be present in high enough concentration.

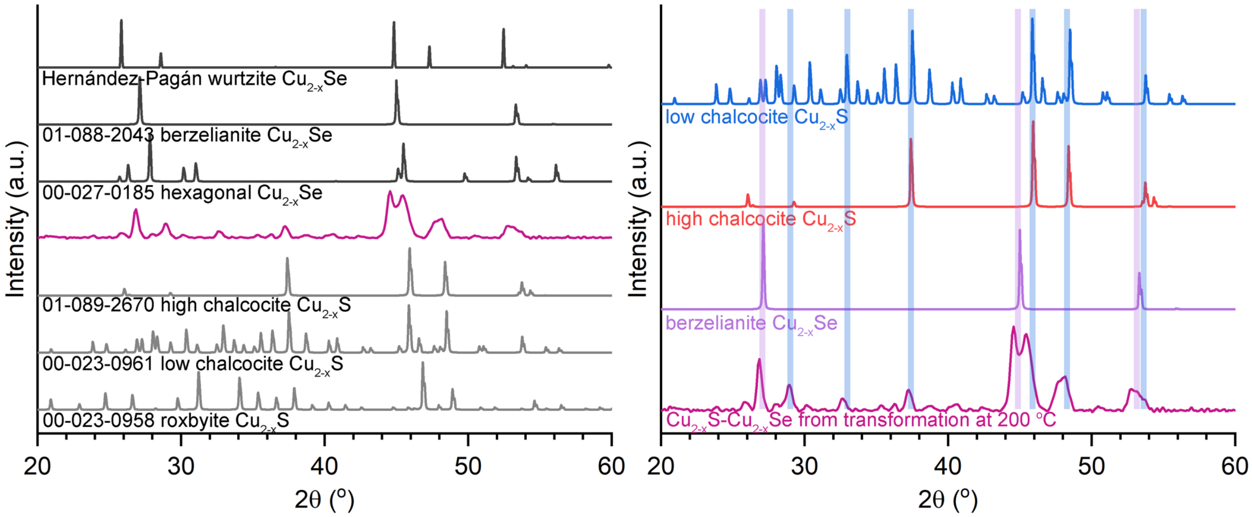

Figure S3. PXRD of $Cu_{2-x}S$ nanorods transformed at 200 °C. a) Comparison with database patterns for various copper sulfide and copper selenide phases, suggesting that it is a mixture of cubic berzelianite copper selenide and a copper sulfide species. b) A closer comparison of the experimental pattern with cubic berzelianite, low chalcocite, and high chalcocite suggesting that low chalcocite is a better match for the copper sulfide present.

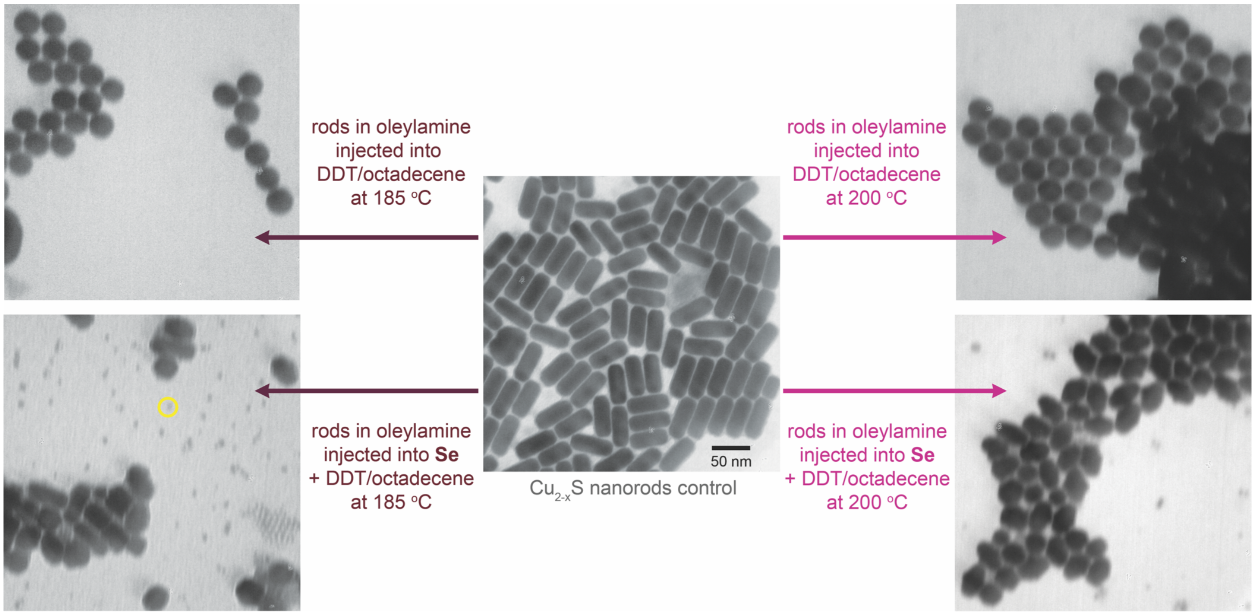

Figure S4. Effect of injecting $Cu_{2-x}S$ nanorods into ddt and octadecene versus Se, ddt, and octadecene at 185 °C and 200 °C. At both temperatures, the rods become spheres in the absence of Se but take on brick shapes at 185 °C and diamond shapes at 200 185 °C when Se is present. Additionally, the presence of Se results in the production of tiny additional particles (an example is shown in yellow) indicative of dissolution processes occurring.

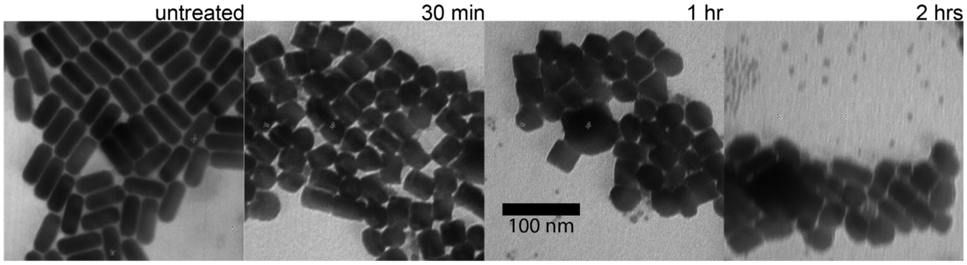

Figure S5. TEM of $Cu_{2-x}S$ nanorods transformed at 185 °C for various times showing a transformation that starts with spherical and brick-like particles and becomes more brick-like over time.

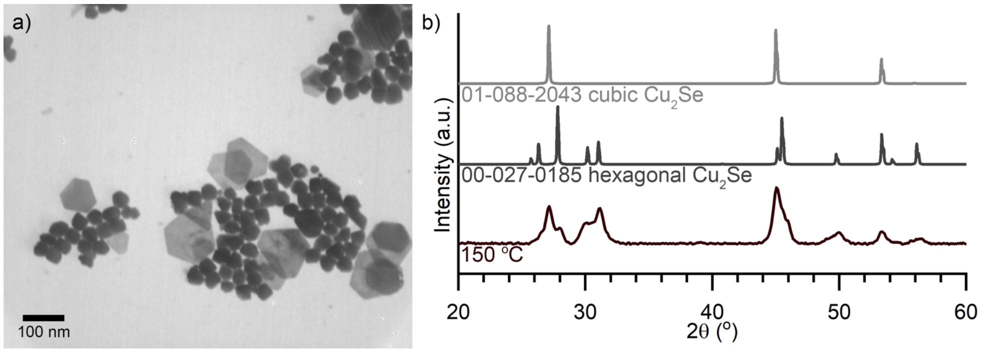

Figure S6. $Cu_{2-x}S$ nanorods in oleylamine were injected into Se, ddt, and octadecene at 150 °C and reacted for 2 hours. a) TEM of the resulting mixture of particles shows many large hexagonal plates as well as smaller facetted particles, suggesting the growth of new, large particles. b) PXRD shows that in addition to the cubic Cu_2Se phase observed at 185 °C and 200 °C, there is also a hexagonal Cu_2Se phase.

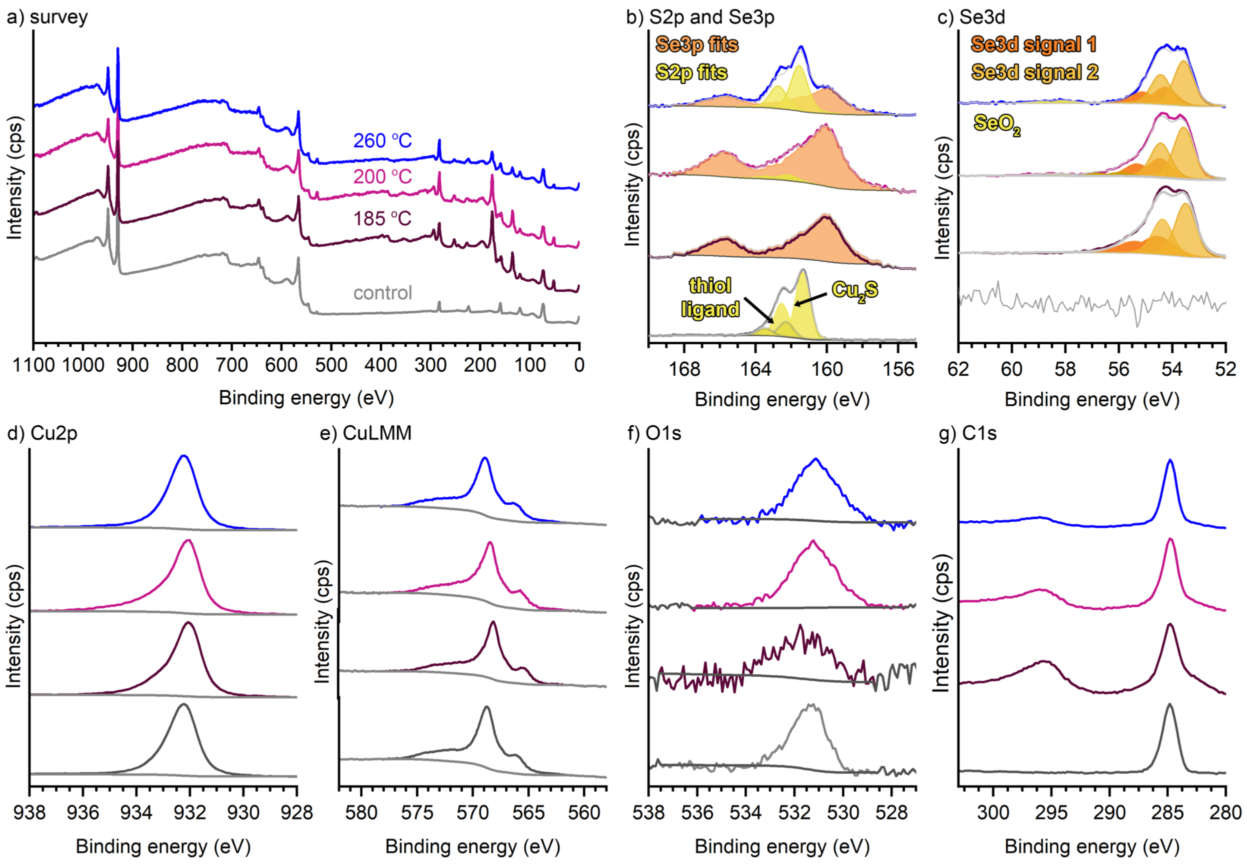

Figure S7. XPS of $Cu_{2-x}S$ nanorods transformed into $Cu_{2-x}S$-$Cu_{2-x}Se$ nanoheterostructures at various temperatures (top, blue 260 ºC, bright pink = 200 ºC, dark pink = 185 ºC, gray = control). The Cu2p (d), CuLMM (e), and O1s (f) signals show no noticeable differences in the oxidation levels or oxidation state of Cu. The survey (a) and C1s (g) show the presence of the same elements with the addition of Se for the transformed samples. The S2p/Se3p (b) region shows significant differences in the S/Se ratio. The control sample shows S2p signal from $Cu_{2-x}S$ and the dodecanethiol ligand. The 185 ºC and 200 ºC samples show Se3p signals overwhelming any small amount of S2p present. The 260 ºC sample has a more balanced amount of S2p and Se3p, but no ligand signal can be discerned.

Before Se^{2-} exchange, the $Cu_{2-x}S$ particles were slightly copper-deficient (as demonstrated by their plasmonic behavior) but the Cu/anion ratio was still very close to 2:1. The copper-deficiency

increases significantly when Se^{2-} exchange occurs at both 185 °C and 260 °C. Here and in the tellurization process(Garcia-Herrera, L. F.; McAllister, H. P.; Xiong, H.; Wang, H.; Lord, R. W.; O'Boyle, S. K.; Imamovic, A.; Steimle, B. C.; Schaak, R. E.; Plass, K. E. Multistep Regioselectivity and Non-Kirkendall Anion Exchange of Copper Chalcogenide Nanorods. Chem. Mater. 2021, 33 (10), 3841–3850. DOI: 10.1021/acs.chemmater.1c01107.), anion exchange of copper sulfide nanorods results in highly Cu-deficient species. Presumably this allows the lattice to accommodate incorporation and transport of larger anions into the copper sulfide lattice. Such a change in atomic rations must be accompanied by oxidation state changes, but they are not obvious in the XPS data (Figure S7). The Cu2p peak is not significantly shifted in the Se^{2-} exchanged sample (Figure S7d) and while there are two Se environments obvious in the Se3d region (FigureS7c), the differences at different transformation temperatures (and degrees of copper-deficiency) is slight. Similarly small effects have been seen when comparing copper sulfides from Cu_2S to CuS (see Xie, Y.; Riedinger, A.; Prato, M.; Casu, A.; Genovese, A.; Guardia, P.; Sottini, S.; Sangregorio, C.; Miszta, K.; Ghosh, S.; Pellegrino, T.; Manna, L. Copper Sulfide Nanocrystals with Tunable Composition by Reduction of Covellite Nanocrystals with Cu+ Ions. *J. Am. Chem. Soc.* **2013**, *135* (46), 17630–17637. DOI: 10.1021/ja409754v.)

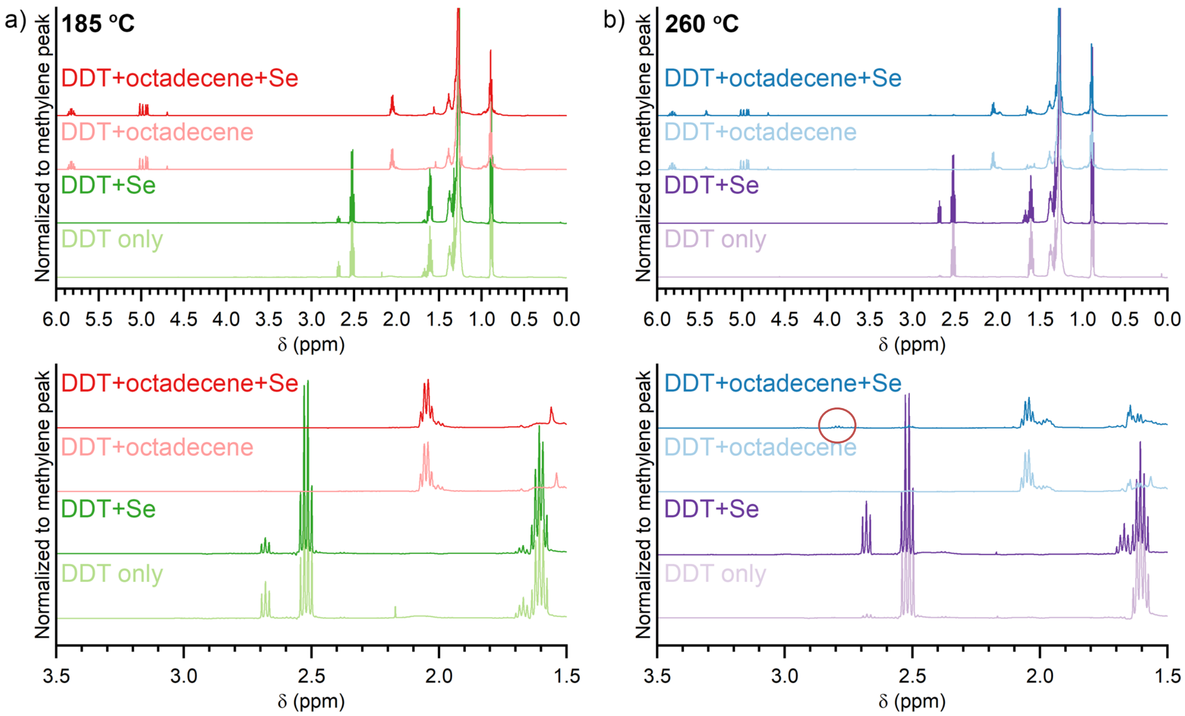

Figure S8. ^{1}H NMR of various reaction mixtures heated at a) 185 °C or b) 260 °C. Top) Full scale spectra of dodecanethiol heated with or without Se and/or octadecene. Bottom) Zoomed in spectra showing the region in which alkyl sulfides and alkyl selenides would appear. Didodecyl diselenide (circled) appears in small yield only when ddt is heated with both octadecene and Se.

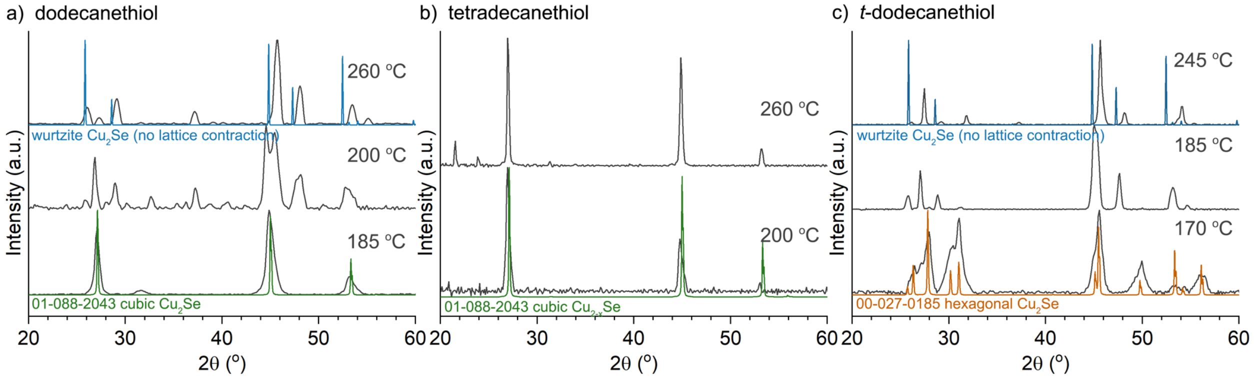

Figure S9. Comparison of PXRD of $Cu_{2-x}S$ nanorods transformed by injection into a solution of Se, octadecene, and various thiol species. a) Dodecanethiol is typically what was used and shows 3 different nanoheterostructures at 185, 200, and 260 ºC. b) Use of tetradecanethiol results only in large particles (as indicated by the sharp peaks) of cubic $Cu_{2-x}Se$ suggestive that $Cu_{2-x}S$ particles are dissolved and $Cu_{2-x}Se$ grows rapidly. c) Use of *t*-dodecanethiol results in a different phase of hexagonal Cu_2Se at low temperatures but forms wurtzite Cu_2Se at 185 ºC and a wurtzite $Cu_2(S,Se)$ solid-solution at 245 ºC. This data indicates that the nature of the alkylthiol species has an important role in influencing the transformation of $Cu_{2-x}S$ nanorods.
